# Diagnosis of compliance of health care product processing in Primary Health Care^1^

**DOI:** 10.1590/1518-8345.1439.2820

**Published:** 2016-11-21

**Authors:** Camila Eugenia Roseira, Darlyani Mariano da Silva, Isis Pienta Batista Dias Passos, Fabiana Souza Orlandi, Maria Clara Padoveze, Rosely Moralez de Figueiredo

**Affiliations:** 2 MSc, Teacher, Centro Estadual de Educação Tecnológica Paula Souza. São Carlos, SP, Brazil.; 3 Undegraduate student in Nursing, Universidade Federal de São Carlos, São Carlos, SP, Brazil.; 4 Doctoral student, Departamento de Enfermagem, Universidade Federal de São Carlos, São Carlos, SP, Brazil.; 5 PhD, Adjunct Professor, Departamento de Gerontologia, Universidade Federal de São Carlos, São Carlos, SP, Brazil.; 6 PhD, Professor, Escola de Enfermagem, Universidade de São Paulo, São Paulo, SP, Brazil.; 7 PhD, Associate Professor, Departamento de Enfermagem, Universidade Federal de São Carlos, São Carlos, SP, Brazil.

**Keywords:** Primary Health Care, Sterilization, Indicators of Health Services, Nursing, Process Assessment (Health Care)

## Abstract

**Objective:**

identify the compliance of health care product processing in Primary Health Care and assess possible differences in the compliance among the services characterized as Primary Health Care Service and Family Health Service.

**Method:**

quantitative, observational, descriptive and inferential study with the application of structure, process and outcome indicators of the health care product processing at ten services in an interior city of the State of São Paulo - Brazil.

**Results:**

for all indicators, the compliance indices were inferior to the ideal levels. No statistically significant difference was found in the indicators between the two types of services investigated. The health care product cleaning indicators obtained the lowest compliance index, while the indicator technical-operational resources for the preparation, conditioning, disinfection/sterilization, storage and distribution of health care products obtained the best index.

**Conclusion:**

the diagnosis of compliance of health care product processing at the services assessed indicates that the quality of the process is jeopardized, as no results close to ideal levels were obtained at any service. In addition, no statistically significant difference in these indicators was found between the two types of services studied.

## Introduction

Health care products manufactured from raw material that permits repeated cycles of cleaning, preparation, disinfection or sterilization can be processed until their functionality is lost. This process should be executed by qualified professionals and includes functionality and quality tests, guaranteeing that the transmission of microorganisms through this route is prevented^1^.

The practice of health care product processing is guided by regulatory policies that are based on risk management and public health safety^2^ ^-^ ^3^. These recommendations differ among countries though, being more or less restrictive, in view of technical-operational, economic, environmental, legal and political issues^2^. In Brazil, this theme is regulated by RDC 15, a resolution by the Brazilian Health Surveillance Agency - ANVISA^1^. 

Studies on the processing of health care products have identified that the quality of this procedure can interfere in patient safety, as it can take place ineffectively, whether in or outside the hospital^4^ ^-^ ^5^, demonstrating infection outbreaks associated with the use of health care products, involving distinct microorganisms in different health care scenarios^2^ ^,^ ^6^.

Considering the expansion and diversification of extra-hospital care, including Primary Health Care (PHC), deeper knowledge is needed on health care product processing in this care context. 

A study developed at rural health services in Nepal describes that 72% of the health professionals interviewed reported using unprocessed equipment and 50% did not have appropriate autoclaves at their services, illustrating the need to improve the infection control practices in PHC^7^.

Actions undertaken in PHC and the availability of health care products for single use or processing vary according to the country's level of economic development, making it difficult to elaborate general recommendations for their processing.

Concerning the Brazilian standards, the recommendations are hardly specific for PHC, except for the classification of the Material Storage Centers (MSC), which in this case can be classified as class 1 MSC^3^. The standardized analysis of data on the situation of health care product processing in Brazilian PHC is undoubtedly highly relevant to expand the knowledge on the theme, guide educational and supervisory policies and to serve as a reference for countries with similar development levels.

Another aspect to be taken into account in this study is a possible difference in the quality of the product processing, depending on the type of service analyzed. In Brazil, Primary Health Care Services working in the traditional model (UBS) and Services working in the Family Health Strategy (USF) constitute PHC^8^. 

Both have particular characteristics, related to human and structural resources. Studies that analyze these services^9^ ^-^ ^10^ suggest that, in terms of functionality, the USF are better assessed, despite problems in terms of infrastructure. 

This study aimed to identify the compliance of the health care product processing in a sample of PHC services, using a specific validated tool^3^, and to assess possible differences in the compliance rates observed between the services characterized as UBS and USF.

## Method

Quantitative, observational, descriptive and inferential study. The objective was to identify the compliance index (CI) of health care product processing at ten PHC services in an interior city in the state of São Paulo.

The city under analysis has 221,950 inhabitants^11^, offering 29 health services in PHC, with 15 USF and 14 UBS, distributed across five Regional Health Administrations (ARES), which coordinate the services within their area.

Convenience sampling was used (randomly using Microsoft Excel(r) 2010) to define the number of services, with a view to including two representatives (one USF and one UBS) from each of the five ARES in the city, resulting in a sample of ten health services (34.5% of all PHC services in the city).

One of the authors collected the data between January 22^nd^ and July 23^rd^ 2013, using a previously validated tool^3^ to assess the health care product processing in PHC. The tool^3^ assesses the structure, process and outcome and is organized by indicators in these three dimensions.

Departing from these three dimensions, the assessment was based on Donabedian's model, which is widely used in health, concerning the assessment of service quality based on health indicators in different contexts^12^ ^-^ ^14^.

As structure indicators, the assessment indicator of the technical-operational resources for the cleaning of health care products (L.1) was used, consisting of 22 items, and the assessment indicator of the technical-operational resources for the preparation, conditioning, disinfection/sterilization, storage and distribution of health care products (PE.5), including 21 items. To assess the process, the assessment indicator of the cleaning process of health care products was used (L.2), with 13 items, and the assessment indicator of the preparation, conditioning, disinfection/sterilization, storage and distribution process of health care products (PE.6), consisting of 36 items. For the outcomes dimension, the assessment indicator of the preserved packing of sterilized health care products (PE.9). 

Each indicator consists of different components, whose information can be obtained by inspection, registration or interview with the responsible professional, depending on their relevance according to the indicator instructions. 

The compliance index of each indicator is obtained by calculating the number of components in compliance/components assessed, divided by the total number of components, expressed in percentage, with 100% as the ideal score. 

For PE. 9, the compliance index is obtained by the number of packages of sterilized health care products with preservation problems divided by the total number of packages, expressed in percentages, with 0% as the ideal compliance index.

Some components required the inspection of a sample of processed products. To calculate this sample, the software OpenEpi(r) was used, totaling 384 items. Therefore, 38 items should be observed at each of the ten health services studied. Due to the varying frequency of the products' use at the health services, this number could not be reached in all situations though, as presented in Table 1. 

**Table 1:** Sample of health care products analyzed to assess the compliance index of the health care product processing quality, according to the processing steps and service type. São Carlos, SP, Brazil, 2013

Step of health care product processing	UBS^*^	USF^†^	Total
Pre-Cleaning	202	200	402
Pre-disinfection products	199	209	408
Immersion of health care products in disinfecting solution	305	252	557
Rinsing of health care products after disinfection	264	210	474
Drying and storing of disinfected health care products	293	275	559
Pre-sterilization of health care products by damp heat	249	185	418
Integrity of packing of stored health care products	237	215	452

* Primary Health Care Service

† Family Health Service

The data were analyzed in the Statistical Package for the Social Sciences (SPSS^(r))^ version 22.0. The descriptive analysis was developed according to the type of service (UBS/UFS). The Mann-Whitney test was applied to compare the quality indicators in the structure, process and outcome indicators between the UBS and USF, considering a 5% significance level (p-value ≤ 0.05). 

Approval for the research was obtained from the Ethics Committee for Research involving Human Beings at Universidade Federal de São Carlos, Opinion 112.528. To apply the indicators that demanded an interview, the person responsible for the processing at the MSC of each service was invited to participate in the study, receiving explanations on the research objective and the Informed Consent Form for signing. All professionals invited agreed to participate in the study.

## Results

During the period observed, the processing at the services studied involved 46 professionals at the UBS (21.7% oral health aids and 78.3% auxiliary nurses or nursing technicians) and 18 professionals at the USF (33.3% oral health aids and 66.7% auxiliary nurses or nursing technicians).

The results concerning the CI of the indicators assessed are displayed in Table 2. In this table, the behavior of the structure indicators L.1 and PE.5, of the process indicators L.2 and PE.6 and of the outcome indicator PE.9 can be observed comparatively in the PHC contexts, based on the compliance index of each.

**Table 2:**
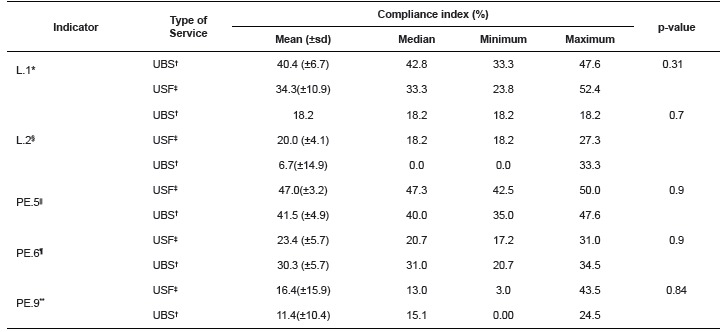
Descriptive analysis and p-value of compliance indices according to the structure, process and outcome indicators of health care product processing and type of service (UBS and USF). São Carlos, SP, Brazil, 2013

* Indicator of technical and operational resources for health care product cleaning

† Primary Health Care Service

‡ Family Health Service

§ Indicator of the health care product cleaning process

| ^|^ Indicator of technical and operational resources for the preparation, conditioning, disinfection, sterilization, storage and distribution of health care products

¶ Indicator of preparation, conditioning, disinfection, sterilization, storage and distribution process of health care products

** Outcome indicator for preservation of packages of sterilized health care products

Concerning the structure indicators L.1 and PE.5, it is added that most health services (60% of UBS and 80% of USF) did not have an exclusive room for expurgation or preparation, conditioning, disinfection, sterilization, storage and distribution of health care products (40% for UBS and 80% for USF). The technical barrier concept was applied at three UBS and two USF.

Regarding L.1, as for the material resources available at the expurgation room, it was observed that 40% of the UBS and 80% of the USF had an appropriate recipient for the disposal of piercing and cutting material; 40% of the UBS and 20% of the USF had deep sinks for product cleaning and 20% of the UBS had a soft brush for this purpose, which was not found at the USF. It is highlighted that, at the other places, steel sponges or toothbrushes were found for this processing step. No individual protection equipment (IPE) was found, such as masks, impermeable gowns and glasses, at 100% of the services studied.

During the assessment, at 100% of the services studied, no product cleaning standards and routines were available, not fully complying with L.1. As for the indicator PE.5, the standards for the preparation, conditioning, disinfection/sterilization, storage and distribution of health care products were present at 40% of the UBS but outdated.

The following were unavailable at the health services studied: forced drying devices, validation documents of steam autoclaves, reports of the quality of the water for the autoclaves (PE.5) or documentation on preventive maintenance of sterilization equipment and records on the efficacy of the sterilization process through chemical, physical or biological tests. At all services, sterilized health products are monitored using an indicator tape of the process. Nevertheless, there is no control on the sterilization by autoclaves through biological indicators (PE.6). 

Sinks for hand washing at the room for the preparation, conditioning, disinfection/sterilization, storage and distribution of health care products, recommended by indicator PE.5, were found at 40% of the units. 

Concerning indicator L.2, at all services, cleaning was done manually using enzyme detergents, without any standardization in the dilution, immersion time or change of product being used. The professionals reported that, in the previous year, no type of training or educative action had been offered on this step of the processing and that they did not participate in the definition of the substances to be purchased. 

As verified by indicator PE.6, at the preparation, conditioning, disinfection/sterilization, storage and distribution rooms of health care products, no magnifying glasses were found to inspect the cleaning of the products, no registers on the sterilization/disinfection and no appropriate packing for the health care products. 

The same indicator mentioned revealed that sodium hypochlorite was the product used to disinfect nebulization material. Its concentration was not standardized though, with values ranging between 0.001% and 1% at the different services studied. As for the preliminary drying of the items to be immersed for disinfection, at two USF, this procedure was done; one UBS and two USF fully immersed the products in the disinfecting solution.

Based on PE.6, the use of damp heat was verified as the sterilization method at 100% of the services, using gravitational autoclaves with less than 100 liters of capacity. It was observed that 60% of the UBS and no USF stored the items for sterilization in the autoclave vertically with space between the packages. Except for one UBS, at the other health services, the professionals awaited the drying and cooling of the products sterilized before removing them from the autoclaves.

According to the data obtained based on the application of PE.6, it was verified that no appropriate product storage places were found, in accordance with current recommendations, which can negatively affect the maintenance of their sterility. 

As regards the preservations of packages of sterilized health care products (indicator PE.9), 462 packages wrapped in kraft paper were inspected. Although this packing is not recommended^1^, the conservation condition of the packing was observed. It was verified that 28 packages were stained, 17 ruptured, 11 dirty, six open, four with the tape that kept them closed detached, two stained and ruptured.

Based on the analysis of the p-value, no statistically significant difference was observed between the types of health services concerning any of the indicators used. Overall, the structure analysis (CI< 50%) was found closer to an appropriate CI when compared to the process indicator L.2, which obtained CI < 20%.

## Discussion

### The structure of the health care product processing services

The professionals engaged in the processing of health care products at the services studied had profiles similar to what was found in the literature, which appoints the nursing team (auxiliary nurses and nursing technicians) as the main responsible for this practice, followed by the dental aid in PHC^4^.

As regards the physical structure, no statistically significant difference was found in the CI between UBS and USF (p-value equal to 0.3 and 0.9 for L.1 and PE.5, respectively), and the mean CI were inferior to 50% for both structure indicators applied. As opposed to the expected difference, considering studies that assessed the structure of health services in PHC and identified that the USF had larger problems related to the physical structure, as these services are frequently domestic adaptations^9^ ^,^ ^15^. 

Structurally, according to the legislation in force^3^, MSC in PHC can be classified as class I MSC, so that there is no need for physical separation between the clean and dirty areas of MSC, the use of the technical barrier should be established to impede the contact between the health care products from the different areas. Technical barrier are considered to be behavioral measures by health professionals to prevent cross-contamination between both areas^1^. The application of this concept was not identified at most of the services studied, whose flow of health care products did not follow a one-way sense. 

As observed in this study, a research developed at four MSC in hospitals from Salvador - BA found no resources on site for hand washing^2^, influencing the prevention of recontamination of already processed products^16^ by collecting them from the autoclave before the storage. 

### Compliance of health care product processing as a process

Departing from the manual execution of product cleaning at all services that participated in the research, the use of steel sponges was observed for this practice, differently from the standards in force in the country^1^. The indicator related to cleaning obtained the lowest CI (Table 2), which can deeply compromise the final quality of the process^17^.

As regards the inputs used for cleaning, the inappropriate use of enzymatic detergents was observed. The range of products of this type that exist in the market and the different usage orientations can justify difficulties in training professionals in the area^17^.

Concerning the solutions used for chemical disinfection, the use of sodium hypochlorite was observed, a substance summarily used to disinfect nebulization products in PHC in Brazil^5^ ^,^ ^18^. 

Aspects of concentration and immersion time are linked to the quality of the disinfection and product integrity, such as the presence of toxic residues on the product that was disinfected, particularly inhalation material^3^. The Brazilian Association of Surgical Center, Anesthetic Recovery and Material Sterilization Central Nurses recommends that it should correspond to 10.000ppm (1%), with a product immersion time of 30 minutes or 200 ppm (0.02%) for 60 minutes of exposure^18^.

Besides the concentration, another aspect related to the disinfection of health products that should be observed is its full immersion, so that the entire surface and all channels have contact with the solution and are then carefully rinsed, removing all toxins and irritating residues^16^.

It is highlighted that similar results were found in a study developed at a UBS in São Luís (MA), where the nebulizers were not subject to preliminary cleaning before the disinfection (89.90% of the services) and sodium hypochlorite for disinfection (78.50%)^18^. 

According to the legislation in force, the gravitational autoclaves are permitted when their capacity is inferior to 100 l, the situation found in the study context. The same legislation is not complied with, due to the absence of reports to support the water quality and records of preventive maintenance of the autoclaves^1^. 

What the inappropriate arrangement of the products inside the autoclaves is concerned and the lack of awaiting the cooling time, this practice compromises the circulation of the steam and lukewarm or hot packages should not be placed on surfaces with a temperature inferior to their own, as this can cause humidity in- and outside the packages, compromising the protection barrier properties of the packing^16^.

Nevertheless, an experimental study to identify the maintained sterility of health care products in case of presence of moisture due to steam showed that, when the conditioning and storage conditions were appropriate, the inside of the sterilized boxes was not contaminated after they were withdrawn while not cooled yet and stored for 30 days^19^. Nevertheless, this cannot be extrapolated to other types of packaging nor to a lack of inappropriate manipulation, like without hand washing for example. 

Biological indicators were not used as frequently as recommended by the assessment tools used and chemical indicators were the most used, similar to a study developed in cities in Goiás, in which the chemical indicator was the most used test in 83.8%^20^. This scenario compromises the confidence that the autoclaves available for the processing of health care products are used for the sterilization at these places.

### Preservation of packing of sterilized products

For the storage of the products processed, a consensus exists that it should at least guarantee the integrity of the packing, avoiding tears, dirt or wetting, which was not found in this study. More controlled situations such as air humidity, temperature and specific storage places (such as cupboards or plastic boxes) do not seem to interfere in the maintenance of their sterility^21^ ^-^ ^22^.

Although inappropriate, the use of Kraft paper is still a reality in PHC^4^, as verified in this study.

Although the study comes with limitations, as it does not permit the establishment of cause-and-effect relations among the findings or the generalization of results, it is extremely relevant, as it presents measurable and standardized data. 

## Conclusion

The diagnosis of compliance of health care product processing at the services assessed indicates the commitment of the process quality, as the CI obtained was not close to ideal levels at any service. In addition, there was no statistically significant difference in the quality indicators of structure, process and outcome between the UBS and the USF investigated, as opposed to the initial research hypothesis.

Cleaning indicators obtained worse CI, for structure as well as for process (L.1 < 40% L.2 CI 20%, respectively), which is undoubtedly a source of concern as it seriously compromises the subsequent steps.

This study joins important systemized information on the panorama of health care product processing in PHC, contributing to the expansion of knowledge on one of the pillars of healthcare-related infection control in this environment.
